# The Oxidative Stress Parameters as Useful Tools in Evaluating the DNA Damage and Changes in the Complete Blood Count in Hospital Workers Exposed to Low Doses of Antineoplastic Drugs and Ionizing Radiation

**DOI:** 10.3390/ijerph18168445

**Published:** 2021-08-10

**Authors:** Jasminka Mrdjanović, Slavica Šolajić, Branislava Srđenović-Čonić, Višnja Bogdanović, Karaba-Jakovljević Dea, Nebojša Kladar, Vladimir Jurišić

**Affiliations:** 1 Oncology Institute of Vojvodina, Faculty of Medicine, University of Novi Sad, 21204 Sremska Kamenica, Serbia; mrdjanovic.jasminka@onk.ns.ac.rs (J.M.); tursijan.slavica@onk.ns.ac.rs (S.Š.); bogdanovic.visnja@onk.ns.ac.rs (V.B.); 2 Department of Pharmacy, Faculty of Medicine, University of Novi Sad, 21000 Novi Sad, Serbia; branislava.srdjenovic-conic@mf.uns.ac.rs (B.S.-Č.); nebojsa.kladar@mf.uns.ac.rs (N.K.); 3 Department of Physiology, Faculty of Medicine, University of Novi Sad, 21000 Novi Sad, Serbia; DEA.KARABA-JAKOVLJEVIC@mf.uns.ac.rs; 4 Faculty of Medical Sciences, University of Kragujevac, 34000 Kragujevac, Serbia

**Keywords:** occupational exposure, antineoplastic drugs, catalase, complete blood count, ionizing radiation, micronuclei, oxidative stress, health risk

## Abstract

Hospital workers at the Oncology Department are occupationally exposed to antineoplastic drugs (ANTNP) or low doses of ionizing radiation (Irrad). Therefore, the aim of this study was to evaluate the level of DNA damage, the oxidative stress parameters and complete blood count (CBC) of hospital workers in order to analyze the negative health effects of ANTNP and low dose Irrad. The frequency of micronuclei (MN) and proliferation index (PI) were analyzed by cytokinesis-block test. The oxidative stress biomarkers evaluated were the level of lipid peroxidation in plasma and catalase activity (CAT) in erythrocytes. A group of 86 hospital workers (35 exposed to ANTPN and 51 to Irrad) had increased MN frequency, CAT activity and level of lipid peroxidation compared to the control group, which consisted of 24 volunteers. The hemoglobin level was lower in the ANTNP group compared to thecontrol group, while a significant difference in RBC was recorded between thecontrol and Irrad groups, and in platelet count betweentheIrrad and ANTNP group. The results showed increased DNA damage, oxidative stress parameters, as well as impairment on complete blood count in hospital workers occupationally exposed to antineoplastic drugs and low-dose ionizing radiation. As this research has shown the importance of oxidative stress, we suggest that in addition to routine methods in periodic medical evaluation, the possibility of applying oxidative stress parameters is considered. Moreover, hospital workers exposed to ANTNP and Irrad in the workplace should undergo not only a more complete health prevention procedure but also have a more appropriate health promotion.

## 1. Introduction

It has been known for over 40 years that exposure to chemical and physical agents increases the risk of developing malignancy. Many studies concerning occupational exposure to ANTNP and Irrad observed genotoxic effects and consequential adverse health effects [1,2,3]. The risk in workers while handling ANTNP and source of Irrad comes from low-dose exposure over a long period of time. It has been reported that the hospital staff handling ANTNP could develop acute and chronic side effects including liver and kidney damage, damage to the bone marrow, damage to the lungs and heart, skin rashes, infertility and cancer [4,5,6]. Oxidative stress is being increasingly recognized as a possible mechanism in the toxicity and carcinogenesis of most of the ANTNP drugs [7].

On the other hand, during diagnostic and therapeutic procedures, hospital workers are at risk from low, repeated, cumulative doses of radiation, especially if they do not take adequate protective measures. An increased risk of developing cancer, particularly of the immune and hematological systems, as well as the mutations of reproductive cells, may be among the consequences of chronic exposition to ionizing radiation [8,9,10]. The production of ROS triggered by Irrad appears to play a central role in these phenomena mechanism. ROS may damage cell structures and, most importantly, DNA. The whole hematopoietic system is among the most sensitive for the side effects of ANTNP and Irrad [11]. Chronic low dose exposure to ANTNP and Irrad may lead to dose-dependent changes in circulating hematopoietic cells through various mechanisms. Some of them are direct damage of hematopoietic stem cells and bone marrow activity reduction. A study conducted on the incidence of malignancies in hospital workers exposed to Irrad confirmed an increased risk for leukemia years after initial radiation exposure [12]. The long-term low dose occupational exposure to Irrad may induce deleterious health effects to workers also manifesting through alteration of blood chemistry parameters [11,12].

The aim of this study was to determine the impact of occupational exposure of hospital workers to antineoplastic drugs and ionizing radiation on three endpoints: DNA damage, antioxidant status and blood counts. The possible impact of smoking, age and gender on investigated parameters was also evaluated.

According to the above-mentioned, the healthcare professionals’ periodic medical surveillance at Oncology Institute of Vojvodina was conducted. DNA damage analyses, as well as blood examination, were performed as part of the overall assessment of their health. Due to the fact that ANTNP and irradiation can induce ROS and consequently alter antioxidant enzymes and lipid peroxidation process in permanently exposed hospital workers, it was considered reasonable to additionally evaluate, among various oxidative biomarkers, catalase (CAT) in erythrocytes as a representative of antioxidant enzymes and thiobarbituric acid reactive substances (TBARS) in plasma as an estimator of lipid peroxidation process.

## 2. Materials and Methods

### 2.1. The Study Participants

The study included 86 subjects from the Oncology Institute of Vojvodina, who represented an exposed group and 24 individuals as a control group.

The control group consisted of volunteers who were not occupationally exposed to any physical, chemical or biological agents. The subjects from this group were enrolled both from the staff employed in administration who worked in another building, and also among extern population. They were also recruited on the basis of gender, age, smoking and eating habits in order to represent a matching group as close as possible.

Based on the type of occupational exposure, the group of hospital workers was divided into two subgroups.

The first subgroup included hospital staff at the Oncology Department handling the following antineoplastics during their preparation and administration: cyclophosphamide, etoposide, cisplatin and doxorubicin, mitomycin c, 5-fluorouracil, methotrexate, vincristine, carboplatin and paclitaxel. The average number of ANTNP drug administrations was 50–60 per day/per hospital worker. Daily exposure time was 2–6 h depending on the organization of work. This refers to reconstituting powdered or lyophilized drugs and further diluting either reconstituted powder or concentrated liquid forms of ANTNP as well as administering drugs by intramuscular, subcutaneous, or intravenous routes. While working with ANTNP, nurses used personal protective equipment such as protective masks, gloves and protective clothes. Drug reconstituting was performed in a separate room in the laminar flow chamber.

The second subgroup consisted of subjects in the radiotherapy unit, exposed to Irrad on linear accelerators type Varian 2100C (15MV), Varian 600DBX (6MV), simulator localizer (Simulex Evolution, VarianMedical Systems, Crawley, UK), CT-scanner (SIEMENS Healthcare GmbH, Erlangen, Germany), equipment for brachytherapy (Gammamed 12 PLUS (Ir^192^), Varian Medical Systems, Crawley, UK) and X-ray source (PHILIPS ENDURA, Amsterdam, The Netherlands). The daily exposure time was also 2–6 h, depending on the type of activity. This referred to 8–15 CT or 20–35 radiotherapy, or 3–5 brachytherapy, or 8–10 tele-radiotherapy, or 1 endoluminal radiotherapyper day/per worker. During the devices’ activities, the workers were in a control room, protected from direct source of ionizing radiation.

The biological monitoring is performed every year, which is regulated by law for the occupationally exposed person, while monitoring of the working environment is done periodically at the request of the employer.

Each participant in this study filled out the questionnaire that included information about smoking habits, alcohol consumption, medical history, drug intake and diagnostic medical irradiation. Persons who had medical treatment, radiography or vaccination within the previous nine months were not included in the study. Moreover, informed written consent was obtained from all of the participants before the study. The study was approved by the Ethics Committee, the decision No XXXII-2013/16.

### 2.2. Sample Collections

The blood samples from all participants were collected over a period of four months. 8 mL of heparinized whole blood was sampled by venipuncture from the cubital vein of each participant and used for the analysis of DNA damage, oxidative stress, as well as CBC.

### 2.3. The Micronucleus Analysis

Heparinized blood samples were cultured for test CBMN that was performed by the standard cytogenetic procedure with minor modifications regarding staining, as described previously [13]. Cells were stained with Giemsa (2%) in distilled water with three drops of NH_4_OH for 9 min. At least 1000 cells per sample were analyzed. The monitored values included: frequency of micronuclei and proliferation index. MN frequency was presented as a number of micronuclei per 1000 examined binuclear cells. The proliferation index (PI), which represents a measure of the number of cell cycles that a cell population passes through, was calculated according to Equation (1):

*PI* = (*M*1 + 2*M*2 + 3(*M*3 + *M*4))/*N* (1)

where *M*1–*M*4 represent the numbers of cells with 1–4 nuclei, respectively, and *N* is the total number of scored cells [14]. The prepared material was observed and analyzed by light microscopy (Olympus BX51, Hamburg, Germany).

### 2.4. The Oxidative Stress Analysis

Erythrocytes were used to determine CAT activity, while the level of lipid peroxidation was measured in plasma. The principle of the catalase assay was based on monitoring the H_2_O_2_ decomposition rate at 240 nm and the results were expressed as U/g Hgb [15]. Lipid peroxidation was estimated by the formation of thiobarbituric acid reactive substances (TBARS) and the results were expressed as nmol of MDA/L [16]. Detailed procedures for the erythrocyte lysate preparation is described in the paper written by Jelić, et al. [17]. For the measurements, Agilent 8453 UV-visible spectrophotometer was used. All chemicals used in this study were of analytical grade and purchased from Sigma Aldrich (St. Louis, MO, USA).

### 2.5. The Complete Blood Cell Count

Complete blood cell count (RBC, WBC, Pl, Hgb) was analyzed by Ektacham 250 hematological analyzer.

### 2.6. The Statistical Analysis

The obtained data were processed by Microsoft Office Excel v2019 (Microsoft Corporation. Redmond, WA, USA), StatsoftStatistica (Statsoft. Tulsa, OK, USA) and R Project for Statistical Computing packages (R Foundation. Vienna, Austria). The dataset included 12 variables and 110 cases. Box plot graphs were used for the data distribution presentation for specific variables, while differences and correlation between the studied groups, as well as variability of samples, were assessed by means of univariate (*t*-test, ANOVA, point-biserial correlation coefficient) and multivariate (MANOVA, principal components analysis for mixed type of data). Differences were considered significant when *p* < 0.05.

## 3. Results

### 3.1. Characteristics of Subjects Included in the Study

The summary of the study participant’s characteristics is presented in Table 1.

### 3.2. The Influence of Occupational Exposure on DNA Damage, Oxidative Stress Parameters and Complete Blood Count

#### 3.2.1. The Influence of Occupational Exposure on DNA Damage

**Figure 1:**
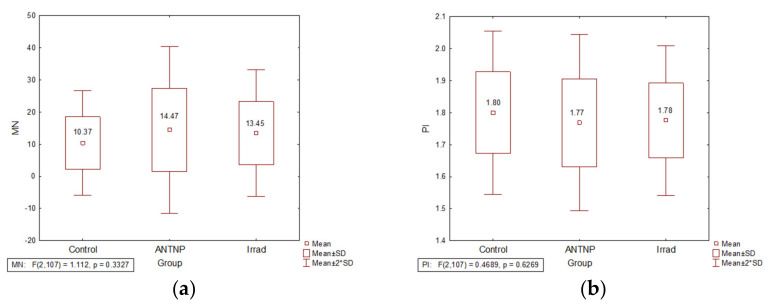
The impact of occupational exposure on DNA damage: (**a**) micronucleus frequency (number of micronuclei per 1000 examined binuclear cells); (**b**) proliferation index.

The frequency of MN in both ANTNP and Irrad hospital workers groups was higher than the values in the control group (ANTNP: 14.77 and Irrad: 13.26 vs. Control: 10.37), without a statistical significance. The MN frequency in the group handling of the ANTNP was higher than in the group exposed to Irrad (Figure 1a). The proliferation index values (PI) were inversely proportional to the MN frequency (Figure 1b).

#### 3.2.2. The Influence of Occupational Exposure on Oxidative Stress Parameters

**Figure 2:**
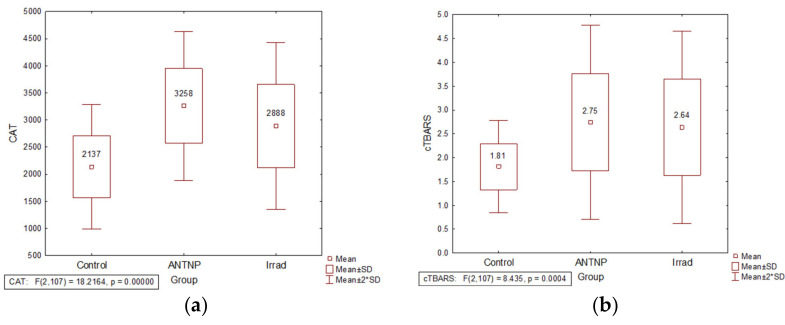
The impact of occupational exposure on oxidative stress parameters: (**a**) catalase activity (U/g Hgb); (**b**) cTBARS (nmol of MDA/L). Control—unexposed volunteers; ANTNP—workers exposed to antineoplastic drugs; Irrad—workers exposed to ionizing irradiation.

The activity of CAT was significantly increased in the erythrocytes of the exposed groups in comparison to the controls (t(57) = −6.56, *p* = 0.000 for ANTNP group and t(73) = −4.26, *p* = 0.000 for Irrad group). CAT activity values were significantly higher in the group exposed to antineoplastic drugs than in that exposed to irradiation (t(84) = −2.29, *p* = 0.024) (Figure 2a).The plasma MDA level was significantly increased in healthcare workers occupationally exposed to antineoplastic drugs (t(57) = −4.174, *p* = 0.000) or ionizing radiation (t(73) = −3.79, *p* = 0.000) when compared to the control group (Figure 2b).

#### 3.2.3. The Influence of Occupational Exposure on Complete Blood Count

The analysis of complete blood count in the exposed and control group showed that Hgb concentration was lower in the ANTNP group when compared to the values in the control group. Furthermore, a significant difference in RBC was recorded between the control and Irrad group and platelets count between Irrad and ANTNP group (Table 2).

#### 3.2.4. Multivariate Statistical Approach

**Figure 3:**
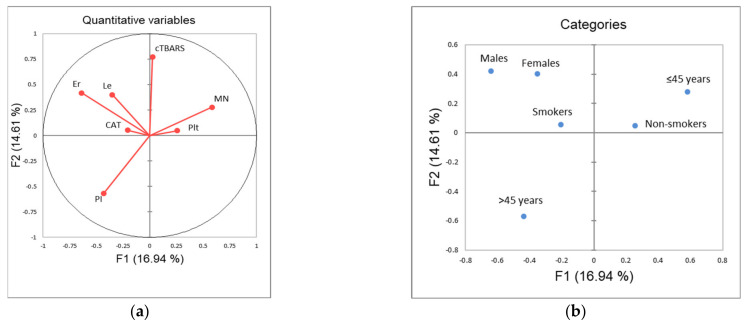

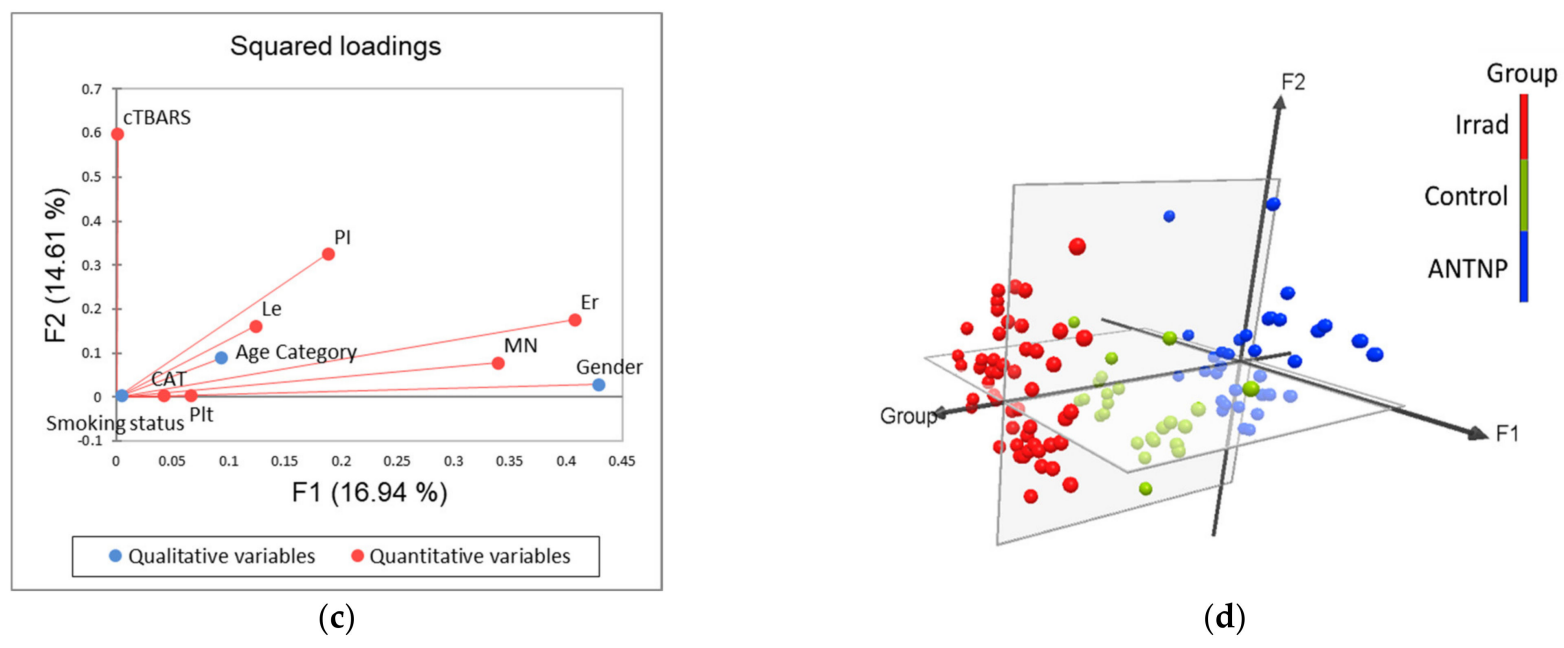
Principal component analysis: (**a**) correlation circle (quantitative variables); (**b**) factorial map of categories; (**c**) squared loadings of all evaluated variables; (**d**) position of evaluated cases in the space defined by the first two principal components. Control—unexposed volunteers; ANTNP—workers exposed to antineoplastic drugs; Irrad—workers exposed to ionizing irradiation.

The application of principle component analysis (PCA) on dataset described by quantitative variables MN, PI, CAT, TBARS, CBC (WBC, RGB, Plt), as well as qualitative variables: gender, smoking status and age category (≤45 years, >45 years), showed that the first two principal components (F1 and F2) describe more than 31% of samples variability (Figure 3). It can be noticed than in terms of F1 the most of the samples’ variability correlates with the gender distribution among the evaluated workers, as well as the incidence of MN and the number of red blood cells (Figure 3c). On the other hand, the shape of the variability (in term of F2) is mostly determined by the measured levels of TBARS. The position of the evaluated samples in the space defined by the first two principal components (Figure 3d) shows, in general, no separative grouping of workers regarding their group designation. In term of F1, a similar distribution of samples on both sides of axes can be observed as a consequence of equally distributed gender value among the evaluated groups, and high variability recorded for MN incidence and red blood cells count. On the other hand, in term of F2, it can be noticed that workers from control group are predominantly located in the negative part, as a consequence of lower recorded TBARS values.

### 3.3. The Influence of Smoking, Age, Gender and Exposure Time

#### 3.3.1. The Influence of Smoking on DNA Damage, Oxidative Stress Parameters and Complete Blood Count

**Figure 4:**
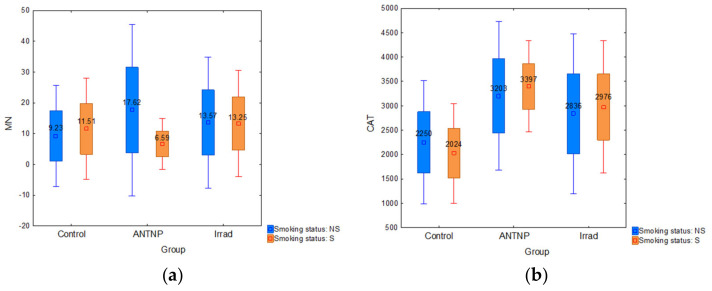

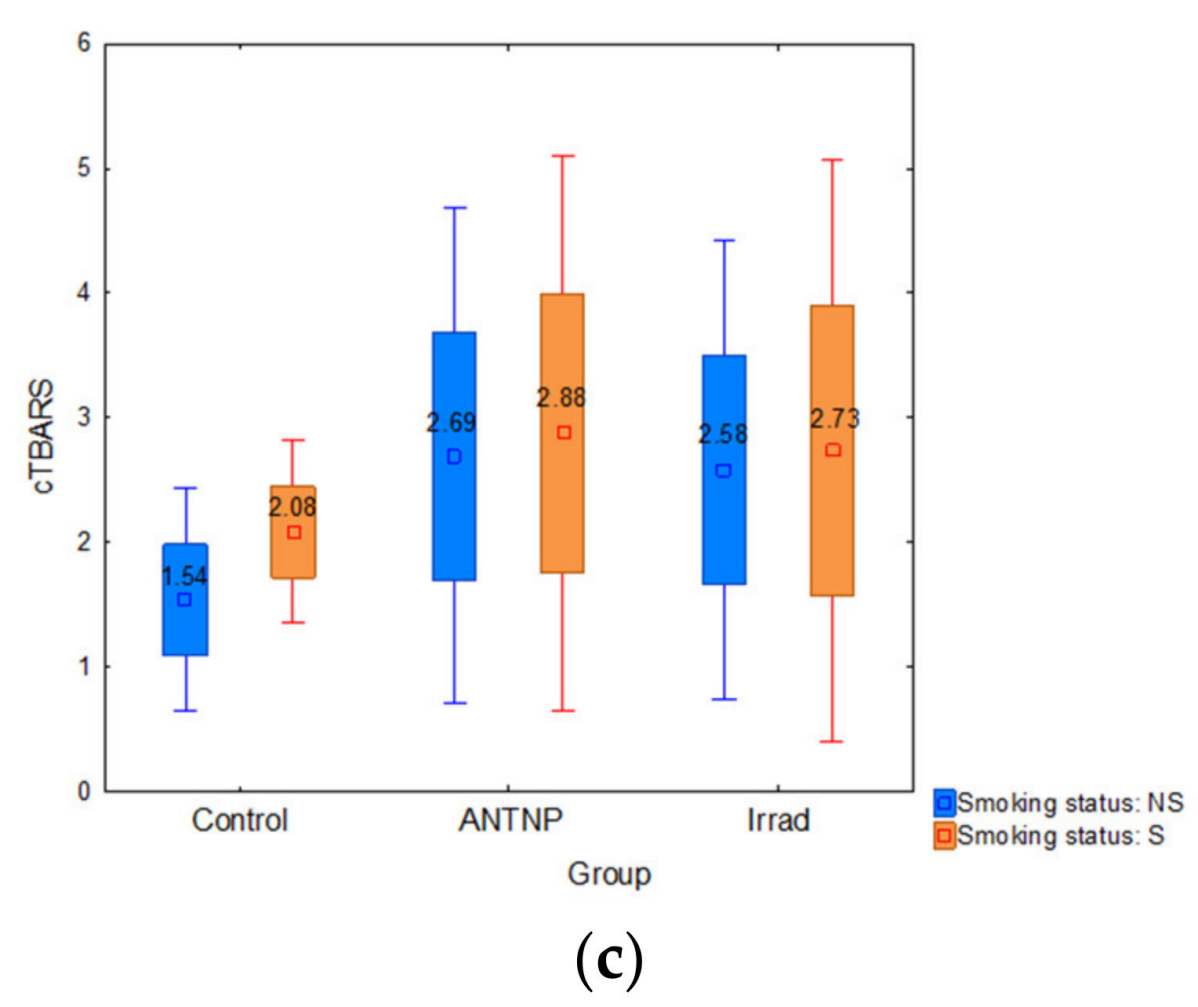
The impact of smoking: (**a**) micronucleus frequency (number of micronuclei per 1000 examined binuclear cells); (**b**) catalase activity (U/g Hgb); (**c**) cTBARS (nmol of MDA/L).

The analysis of smoking as a confounding factor showed that the smokers in the control group had higher MN frequency than nonsmokers (MN: 11.51 ± 8.22 vs. 9.23 ± 8.25) (Figure 4a). There is no statistically significant influence on the level of CAT (Figure 4b) and lipid peroxidation activity (Figure 4c) within the ANTP (F(2, 32) = 0.43, *p* = 0.65) and Irrad (F(2, 48) = 0.33, *p* = 0.72) groups. The statistically significant alteration of cTBARS was observed only in the control group (F (2, 21) = 5.10, *p* = 0.016). cTBARS values in both groups of exposed workers were slightly higher in smokers compared to nonsmokers. According to the results, smoking does not have a significant influence on the complete blood count parameters (WBC, RGB, Plt and Hgb) in the control group (F(4, 19) = 1.1045, *p* = 0.3832), ANTNP group (F(4, 30) = 0.13759, *p* = 0.9671), nor in Irrad group (F(4, 19) = 1.1045, *p* = 0.3832). Smokers tended to have higher leukocyte counts than non-smokers, although without statistical significance.

#### 3.3.2. The Influence of Age and Gender on DNA Damage

**Figure 5:**
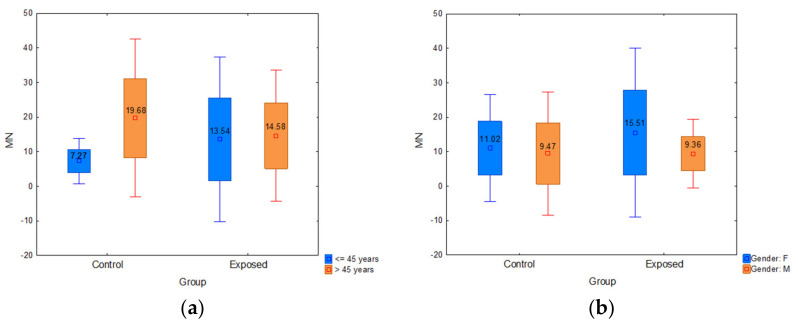
(**a**) Influence of participants’ age on micronucleus frequency; (**b**) influence of participants’ gender on micronucleus frequency. Control—unexposed volunteers; exposed—workers subjected to antineoplastic drugs or ionizing irradiation.

The participants were divided into two subgroups according to age: the younger ones (≤45 years) and the older ones (>45 years). The control group was composed of 18 younger vs. six older subjects, while the exposed group counted 56 younger vs. 28 older ones. Regarding age, older participants had significantly higher MN frequency than younger ones in the control group (*p* = 0.000) and in the exposed group, older workers had a slightly higher median value of MN frequency than younger ones (Figure 5a). According to the gender, participants were divided into male and female groups (Table 1). The MN frequency was higher in female participants compared to males in both control and exposed groups. Moreover, female workers had significantly higher MN frequency (Figure 5b) than males (16.61 vs. 13.42; *p* = 0.022).

#### 3.3.3. The Influence of Exposure Time on Oxidative Stress Parameters

**Figure 6:**
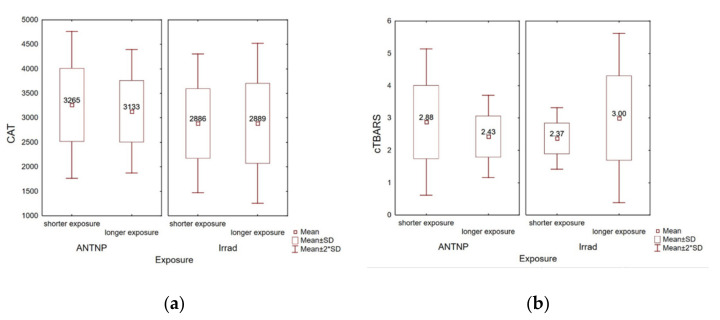
Correlation between the exposure time at work (in years) and oxidative stress parameters. (**a**) Catalase activity (U/g Hgb); (**b**) cTBARS (nmol of MDA/L).

The hospital workers were divided into two subgroups according to the exposure time: workers with shorter (ANTNP: 1–14 years, Irrad: 1–17 years) and longer (ANTNP: 15–30 years, Irrad: 18–36 years) exposition time. No statistically significant correlation was noticed within the subgroups of workers exposed to ANTNP regarding measured CAT levels (r_pb_(35) = −0.09, *p* = 0.60) or cTBARS (r_pb_(35) = −0.21, *p* = 0.21) (Figure 6a). On the other hand, within the Irrad group, although no statistically significant correlation was noticed between the exposure time and CAT (r_pb_(51) = 0.00, *p* = 0.99), the levels of measured TBARS significantly positively correlated with the worker’s exposure time (r_pb_(51) = 0.31, *p* = 0.02) (Figure 6b).

## 4. Discussion

In this study, we provided data on the association between occupational exposure and the extent of primary DNA damage evaluated by the micronucleus test, oxidative stress parameters, as well as the complete blood count in hospital workers occupationally exposed to antineoplastic drugs and ionizing irradiation at the Oncology Department. Likewise, the impacts of additional factors on the examined biomarkers, such as smoking status, age, gender of participants and exposure timehave also been analyzed.

### 4.1. DNA Damage

ANTNP represents a heterogeneous group of chemicals, whichincludes cytostatic drugs, hormones and antibiotics, all of which have a well-known characteristic to inhibit cancer growth. Each group has a different activity mechanism, so, for example, cytostatic drugs may act as alkylating agents, antimetabolites and mitotic inhibitors, free radical generators with strong oxidative properties and topoisomerase II inhibitors.This results in a different type of DNA damage in both normal and cancer cells, including mutation and/or cell death. The occurred DNA damage may be repaired, miss repaired, or not repaired, thus triggering cell transformation and representing a health risk [18]. According to the International Agency for Research on Cancer, 11 ANTNP belong to Group 1 (human carcinogens), 9 to Group 2A (probable human carcinogens) and 10 to Group 2B (possible human carcinogens) [19]. Due to the low selectivity to cancerous cells, ANTNP used in cancer therapy also have adverse effects on healthy cells. Although hospital workers are exposed to much lower doses than cancer patients, handling with ANTNP during a long period of time may cause adverse effects on their health [20]. Concerning occupational toxicity, it is important to point out that hospital workers who handled ANTNP are exposed to their mixture and that the drugs could have a synergistic effect. Their different mechanisms of action imply their different potential to induce genome damage in hospital workers so occupational toxicology represents a wide area of possible harmful effect on the workers’ health [3].

The main potential routes of professional exposure to ANTNP are inhalation and skin or mucosa adsorption during preparation and administration of therapy and cleaning of dust and spillage caused by tablets breakage. The uptake of the ANTNP in the exposed workers was confirmed by detecting the parent molecules and/or their metabolites in the urine [21]. Because occupational exposure to ANTNP implies the handling of multiple drugs, the threshold dose cannotbe clearly determined to identify their combined genotoxic and carcinogenic effects.

In our study, we noticed the insignificantly increased DNA damage in a group of workers exposed to ANTNP in comparison to non-exposed volunteers. The recorded result is in accordance with many studies in which, besides MN test, DNA damage was confirmed by chromosomal aberration, sister chromatid exchange and comet assays [22,23,24]. This result, as well as conclusions of our previous study on the same group of hospital workers [3] could be explained in relation to the protective equipment (gloves, masks, goggles and cabin) that was not used adequately to completely prevent exposure to genotoxic xenobiotics [22].

It is also well known that Irrad can affect DNA directly and/or indirectly via radiolysis of water, thereby creating a reactive oxidative species [14]. ROS generated during the endogenous process, as well as those from working environment, can damage nucleic acids, which is followed by many different types of DNA changes [2]. As a result of raised ROS, transcription factors and their corresponding genes are continuously activated, which, united with the increased DNA damage, creates the environment for the occurrence of carcinogenesis [25]. Taking into account all of the above, individuals occupationally exposed to Irrad with high presence of MN can accumulate mutations and, consequently, develop health problems such as cancer [26]. The health risk will be approximately five-fold higher if the person is exposed over long periods due to a cumulative effect of Irrad [27].

In this study, we examined hospital workers employed in the radiotherapy unit who were occupationally exposed to Irrad at low doses. The results showed that none of the workers exceeded the dose limit of 20 mSv (the average limit for a five-year period according to the International Commission on Radiation Protection) and thus did not reach the average annual effective dose limit of 50 mSv [28]. Despite the fact that the median annual dose in the exposed group did not exceed 2.06 mSv, we revealed increased DNA damage compared to the control group. This observation is in accordance with the results of our previous study, as well as with the literature data for occupationally exposed persons [2,13,27,29]. However, DNA damage noticed in Irrad exposed personnel was lower in comparison to ANTNP exposed.

We have also analyzed the proliferation index, a marker that representing a measure of the lymphocytes mitogen response, immune functions and cytostatic effects of various agents, and general toxicity [30,31]. In our study, the PI values were inversely proportional to the micronucleus frequency since the highest PI values were noticed in the control group and the lowest in the ANTNP group. This appearance was noticed in our previous studies, as well as in other investigations [2,3,13]. Minozzo, Deimling, Gigante and Santos-Mello [31] proposed three hypotheses that could offer the possible reasons for reduced PI value after exposure to ANTNP and Irrad. Namely, the genotoxic agents could cause extensively DNA-damaged circulating lymphocytes die before cell division. Furthermore, the induction of mitotic delay without the DNA reparation could decrease the number of mitoses and consequently PI value. Finally, a clastogenic and an aneugenic effect of genotoxic agents may induce blockade of the cell cycle.

### 4.2. Oxidative Stress Parameters

Occupational exposure to ANTNP and Irrad was also shown to induce ROS and consequently oxidative stress in cells, which commonly brings a response, an alteration in activities of antioxidant enzymes [7,32]. Hence, one of the aims of this study was to monitor oxidative stress-induced in occupationally exposed hospital personnel. CAT and MDA levels have been recognized as relevant oxidative stress markers since they were found to be significantly altered in various pathological conditions, as well as a result of exposure to ANTNP and Irrad [7,17,33]. In our study, the activity of CAT was significantly increased in the exposed groups in comparison to the control, which might represent the adaptive mechanism of the cell since the excessive ROS production selectively increases the transcription of genes coding for antioxidative enzymes, including CAT [34]. Moreover, the plasma MDA level, as a measure of lipid peroxidation, was significantly increased in healthcare workers occupationally exposed to ANTNP and Irrad when compared to the control group. Our findings are in accordance with study of Ahmad, Temme, Abdalla and Zimmerman [10], who concluded that radiation, even within recommended annual dose limits, appears to result in an altered redox balance evidenced by an increase in superoxide anion and lipid peroxidation, accompanied by an increase in SOD activity.

Interestingly, CAT activity values were significantly higher in the group exposed to antineoplastic drugs than those exposed to irradiation. Our assumption is that less precaution is taken in the exposure to the antineoplastic drugs than to the irradiation, leading to the higher degree of oxidative stress, accordingly. Similar conclusion can be noticed in the paper of Mahboob, Rahman, Rekhadevi, Sailaja, Balasubramanyam, Prabhakar, Singh, Reddy, Rao and Grover [7] who suggested that nurses occupationally exposed to ANTNP were susceptible to the oxidative stress and emphasized the need for a harmonized safe handling approach [6,7]. Periodic checks of oxidative stress using biological endpoints such as CAT activity and MDA level to evaluate occupational exposure to ANTNP or Irrad could be of importance to guide health promotion and disease prevention.

### 4.3. Complete Blood Count

Hematologic changes are often used as biological markers for medical surveillance and early detection of health problems connected to occupational exposure to ANTNP and Irrad, which, as stated above, lead to DNA damage and oxidative stress. These underlying mechanisms can affect the number and interrelationships of blood elements and lead to immunosuppression and, consequently, cytopenia [35,36,37,38]. On the other side, there are also other factors contributing to occupationally induced carcinogenesis such as non-targeted effects, inflammation and complex interaction between ionizing radiation, antineoplastic drugs and the immune system [39].

In our study, within the normal range, differences between the group of hospital workers exposed to ANTNP and the control group were found in the number of white blood cells and platelets. According to Tompa, et al. [40], nurses handling cytotoxic drugs had a significantly increased percentage of helper T-cells alongside with significantly elevated Th/Tc ratio. In our study, differential blood cell count was not conducted, so the changes in number of T lymphocytes could not be observed. The authors also pointed out the adverse health effects such as iron deficiency and anemia in exposed workers. Our results also revealed lower hemoglobin concentration in workers exposed to antineoplastic, as well as significant differences in RBC count between control and Irrad groups. When comparing our results to available literature data on radiation workers, it is evident that most of the research conducted on the blood count showed different outcomes. Namely, in a number of studies, no difference was found between the blood parameters of exposed medical workers and the controls [41,42], while some studies reported an increase or decrease in different blood parameters [43,44,45]. Generally, although some of the parameters of CBC tests of occupationally exposed hospital workers were significantly different in comparison to controls, there was no general agreement on specific blood parameters in the assessment of long-term health risks. Healthcare workers are exposed to a large number of concomitant risks, and using the CBC with other complementary methods, such as the evaluation of chromosomal changes and oxidative stress parameters, could be enforced for the early detection of such risks. The application of multivariate statistical analysis on dataset described by variables MN, PI, CAT, TBARS and CBC showed that groups of occupationally exposed hospital workers were separated in relation to the control group. Increased MN frequency, CAT and TBARS values, and significant differences regarding Plt count contributed to the greatest distance of the ANTNP group.

### 4.4. Smoking, Age, Gender and Exposure Time

It is known that cigarette smoking causes deleterious cell damage and is one of the factors which is additionally important in the evaluation of professional exposure. In our study, smokers had higher MN frequency than nonsmokers only in the control group. Since cigarette contains over 69 carcinogenic substances, and several tumor promoters or cocarcinogens [46], the expected increase of DNA damage was noticed. However, many studies confirmed that smoking habits do not affect MN frequency [2,3,13,23,47], as we have seen in both groups of hospital workers. The explanation could be that cells with DNA damage are not included in cell division ex vivo, probably because of disappearance by apoptosis or necrosis [48]. Despite the fact that in large studies regarding smoking habits, a significant correlation was found only in the group of heavy smokers (over 30 cigarettes per day), it is important to keep in mind interaction between smoking habit and occupational exposure to genotoxic agents and always test it [47,48]. Because of their nonspecific nature, CAT and LP may be influenced by outside sources of exposure, especially tobacco smoke. The analysis of smoking as a confounding factor in our investigation showed a statistically significant alteration of oxidative stress parameters only in the non-exposed control group. Based on the previously conducted statistical analyses, it is highly justified to assume that non-significant but enhanced levels of CAT and TBARS in the exposed groups are the consequence solely of workplace exposure to cytotoxic agents or irradiation. Participants’ smoking status did not seem to influence complete blood count in all examined groups. Mean values of RBCs, Platelets, WBCs and HGB, did not differ between smokers and nonsmokers. However, WBC count was increased in subgroup of smokers compared to nonsmokers. It has been shown that smoking has both acute and chronic effect on hematological parameters. A number of studies have reported that smokers of both genders have significantly higher number of leukocytes and elevation in neutrophils, lymphocytes, leukocytes, monocytes, eosinophils and basophils [36]. Malenica, et al. [49] showed severe adverse effects of cigarette smoking on some hematological parameters. Namely, Hgb and WBC levels were significantly higher in smokers compared to nonsmokers, but there was no significant difference in terms of lymphocyte and monocyte counts. When analyzing our results regarding smoking habits of examined subjects and changes in CBC, no significant difference was noticed between smokers and nonsmokers within the control, Irrad and ANTNP groups. However, higher values in WBC count were recorded in smokers in all examined groups. Additionally, tobacco smoking shows harmful effects on WBCs in smokers of both genders. Yanbaeva, et al. [50] explained elevated WBC counts in smokers by a systemic low grade and vascular endothelial inflammatory response.

In addition to the analysis of the occupational exposure impact on the health risk, it is essential to bear in mind the influence of age and gender as factors of undoubted importance on DNA damage. Regarding the impact of age, results in our study tended to rise in older subjects (over 45 years), although with significant extend only in the control group, probably because in the exposed hospital workers, the effect of ANTNP or Irrad was predominant. The increase of MN with age could be explained by a combination of the cumulative effect of acquired mutations in genes involved in DNA repair, chromosome segregation and cell cycle checkpoint. On the other side, chromosomal aberrations caused by the perennial impact of endogenous, environmental or occupational genotoxins, inadequate nutrition, as well as a wide range of other unhealthy lifestyle habits [48] additionally might explain the age-depended rise of MN frequency in our study. In terms of gender impact on DNA damage, our results confirmed that females had higher DNA damage compared to males. The increase of MN frequency in the female gender can be attributed to the greater tendency of the X chromosome to be lost as an MN relative to other chromosomes and to the fact that females have two X chromosomes, as opposed to only one in males [51]. This observation is in accordance with the literature data [2,48] where MN frequency in women is increased 1.2 to 1.6 times than in men [52].

Finally, the exposure time (expressed in years) and oxidative stress parameters correlation analysisrevealed positive result regarding longer exposuretime (18–36 years) of workers in the radiation zone and cTBARS values. This is a particularly interesting result since all employees exposed to Irrad received an average dose of 2.06 mSv during one year at work and thus remained within the reference limits of the permitted radiation dose. This result is in accordance with the previously conducted study [53] and indicates that the analysis of lipid peroxidation process could serve as an additional diagnostic tool for identifyinghospital workers with a higher risk of low dose ionizing radiation.

Taking into account the results of this study, which indicate the fact that even without accidental situations workers exposed to low doses of ANTNP and Irrad had changes in the examined biological parameters, the question arises about possible implications for occupational health surveillance from an ethical perspective. Since the “occupational health”, according to Iavicoli, include not only health protection, but also health promotion in the workplace, it is necessary to have a closer collaboration between the occupational health professionals and employers, workers and their organizations, as well as the competent authorities, professional and scientific associations for implementing the highest standard of ethics in occupational health practice [54].

## 5. Conclusions

This study shows increased DNA damage, oxidative stress parameters, as well as impairment on complete blood count in hospital workers occupationally exposed to antineoplastic drugs and low-dose ionizing radiation. Taking into statistical account all analyzed biomarkers, the ANTNP group was clearly separated in relation to Irrad and especially the control group.

Additionally, this study indicates that smoking as a confounding factor increases the count of leukocytes in all examined groups, while significant alteration of oxidative stress parameters and enhanced level of DNA damage caused by smoking was enhanced only in the control group. The impact of age and gender on examined biomarkers was seen only regarding micronucleus frequency. The increase in oxidative damage to cells with longer exposuretime seen in the group of workers exposed to Irrad, once again emphasized the need for monitoring oxidative stress parameters as useful additional biomarkers.

This highlights the necessity not only for annual monitoring of the occupational exposure biomarkers but also for more adequate implementation of issued safety measures. In order to minimize risks to health and safety, hospitals should ensure workers are adequately trained and educated in the safe use of substances or procedures that they handle. Viewed in the broader context, hospital workers exposed to ANTNP and Irrad should undergo not only a more complete health prevention procedure but also have more adequate health promotion.

## Figures and Tables

**Table 1:** The characteristics of control and both exposed groups.

Variables		Control (*n* = 24)	Exposed		
			All	ANTNP	Irrad
			(*n* = 86)	(*n* = 35)	(*n* = 51)
Age (years)	Median	37.88	40.05	36.43	42.58
	Range	23–68	21–61	21–56	24–61
Gender	Male	10	23	2	21
	Female	14	63	33	30
Smoking status	Non-smokers	12	57	25	32
	Smokers	12	29	10	19
Exposure time (years)	Shorter		7	7	8
	Longer		20	19.25	21
Irradiation during one year (mSv)	Median				2.06
	Range				1.70–3.02

Control—unexposed volunteers; ANTNP—workers exposed to antineoplastic drugs; Irrad—workers exposed to ionizing irradiation. The exposure time data are presented as median values.

**Table 2:** The impact of occupational exposure on complete blood count.

Group	WBC	RGB	Plt	Hgb
	$\bar{X}\;\pm \;\mathrm{SD}$			
Control	7.06 ± 1.42	4.47 ± 0.40	236.87 ± 38.00	138.08 ± 12.58
ANTNP	7.18 ± 1.67	4.63 ^b^ ± 0.38	260.37 ^a^ ± 73.96	130.20 ± 11.67
Irrad	7.06 ± 2.02	4.68 ± 0.39	222.45 ± 45.37	139.88 ± 12.09

^a^ statistically significant differences (t(84) = −2.94, *p* = 0.004) between ANTNP and Irrad group. ^b^ statistically significant differences (t(73) = −2.24, *p* = 0.028) between Irrad and Control group. Control—unexposed volunteers; ANTNP—workers exposed to antineoplastic drugs; Irrad—workers exposed to ionizing irradiation.
